# Ex-Th17 Foxp3+ T cells - a novel subset of Foxp3+ T cells induced in cancer

**DOI:** 10.1186/2051-1426-3-S2-P273

**Published:** 2015-11-04

**Authors:** Stephanie Downs-Canner, Roshni Ravindranathan, Robert P Edwards, Pawel Kalinski, Kunle Odunsi, David L Bartlett, Natasa Obermajer

**Affiliations:** 1 University of Pittsburgh, Pittsburgh, PA, USA; 2 Magee-Womens Research Institute Ovarian Cancer Center of Excellence, Pittsburgh, PA, USA; 3 Roswell Park Cancer Institute, Buffalo, NY, USA

## 

Th17 and regulatory T (T_reg_) cells are integral in maintaining immune homeostasis and Th17-T_reg_ misbalance associates with inflammation.

We demonstrate that in addition to natural (n)T_reg_ and induced (i)T_reg_ cells developed from naïve precursors, Th17 cells are a novel source of Foxp3^+^ cells by converting into ex-Th17 Foxp3^+^ cells, and this helps to reconcile the contradictory information about the relevance in particularly of Th17 subset in immune surveillance.

We identified IL-17A^+^Foxp3^+^ double-positive and ex-IL-17-producing IL-17A^-^Foxp3^+^ T cells to be the underlying mechanism of immune regulation in mesenchymal stem cell-mediated prolonged allograft survival. Further, we identified accumulation of IL17A^+^Foxp3^+^ and ex-Th17 Foxp3^+^ cells in tumor bearing mice, indicating progressive direct Th17-into-T_reg_ cell conversion as a novel phenomenon in cancer.

Moreover, we determined the importance of the Th17 cell plasticity for tumor induction and/or progression in ROR-g^-/-^ mice. Our data indicate that RORgt is required not only for Th17 development, but also for effective T_reg_ cell induction. TGF-b_1_ induced Foxp3 expression was reduced in ROR-g ^-/-^ cells. Further, tumor bearing ROR-g^-/-^ mice showed significantly less Foxp3^+^ T_reg_ cells, but higher IFNg^+^ Tcells compared to wild type animals.

Increased infiltration of IL17^+^ and FoxP3^+^ CD4^+^ T cells in the human ovarian cancer ascites, with the presence of a distinct IL17^+^FoxP3^+^ subset, and a significant correlation between tumor-associated Th17 and T_reg_ cells demonstrates the existence of Th17-Foxp3^+^ T cell inter-relationship in cancer patients.

Yin-yang of IL17^+^ and Foxp3^+^ is important principle for improved clinical approaches targeting responses against self, allo and/or neo-self.

